# Rheumatoid Meningitis Presenting With Acute Parkinsonism and Protracted Non-convulsive Seizures: An Unusual Case Presentation and Review of Treatment Strategies

**DOI:** 10.3389/fneur.2019.00163

**Published:** 2019-02-27

**Authors:** David Pellerin, Michael Wodkowski, Marie-Christine Guiot, Hisham AlDhukair, Andrea Blotsky, Jason Karamchandani, Evelyne Vinet, Anne-Louise Lafontaine, Stuart Lubarsky

**Affiliations:** ^1^Department of Neurology and Neurosurgery, Faculty of Medicine, McGill University, Montreal, QC, Canada; ^2^Division of Rheumatology, Department of Medicine, Faculty of Medicine, McGill University, Montreal, QC, Canada; ^3^Department of Pathology, Faculty of Medicine, McGill University, Montreal, QC, Canada; ^4^Department of Neurology, King Fahad Medical City, Riyadh, Saudi Arabia; ^5^Division of General Internal Medicine, Department of Medicine, Faculty of Medicine, McGill University, Montreal, QC, Canada; ^6^Centre for Medical Education, Faculty of Medicine, McGill University, Montreal, QC, Canada

**Keywords:** rheumatoid arthritis, rheumatoid meningitis, vasculitis, parkinsonism, seizure, immunosuppressant, corticosteroids, rheumatoid granuloma

## Abstract

Rheumatoid meningitis is a rare complication of rheumatoid arthritis (RA). It is associated with substantial morbidity and mortality. The condition may present in a variety of ways and is therefore diagnostically challenging. Uncertainty still exists regarding the optimal treatment strategy. Herein, we describe the case of a 74-year-old man with a history of well-controlled seropositive RA on low-dose prednisone, hydroxychloroquine, and methotrexate. The patient presented with a several-month history of multiple prolonged episodes of expressive aphasia, right hemiparesis, and encephalopathy. Although no epileptiform activity was recorded on repeated electroencephalography, the symptoms fully resolved following treatment with antiepileptic drugs. He subsequently developed acute asymmetrical parkinsonism of the right hemibody. Magnetic resonance imaging revealed subtle enhancement of the leptomeninges over the left frontoparietal convexity. Cerebrospinal fluid analysis revealed a mild lymphocytic pleocytosis and elevated proteins. Histopathologic analysis of a meningeal biopsy revealed nodular rheumatoid meningitis. The patient was treated with corticosteroids and cyclophosphamide, following which he incompletely recovered. This is the first description of rheumatoid meningitis manifesting with acute parkinsonism and protracted non-convulsive seizures. A summary of cases reported since 2005, including data on pathology, therapy and outcomes, along with a discussion on the efficacy of different treatment strategies are provided.

## Introduction

Rheumatoid arthritis (RA) may be associated with various neurological complications, most commonly compressive cervical myelopathy from atlantoaxial subluxation and entrapment neuropathies (1). Direct inflammatory involvement of the central nervous system (CNS) manifesting as meningitis and/or vasculitis has been described in a rare subset of seropositive RA patients (2). Its occurrence appears to be independent of underlying RA disease duration and severity (3). The condition remains diagnostically challenging owing to its highly variable clinical presentation, its nonspecific imaging findings, and the ultimate requirement for histopathological confirmation. Focal neurological deficits, cranial neuropathies, seizures, encephalopathy, and headache are commonly reported presenting features (3, 4). Infrequently, rheumatoid meningitis may mimic common neurological disorders such as transient ischemic attack (5–7) and Parkinson's disease (8, 9). Although no standardized recommendations exist to guide treatment, high-dose corticosteroids alone or in combination with additional immunosuppressive agents are traditionally instituted therapy for this condition. Herein, we describe an unusual presentation of rheumatoid meningitis, manifesting as acute parkinsonism and non-convulsive seizures, occurring in a patient on dual disease-modifying antirheumatic drugs (DMARDs). We also review the efficacy of treatment regimens for rheumatoid meningitis.

## Case Report

### Clinical History

**Figure 1:**
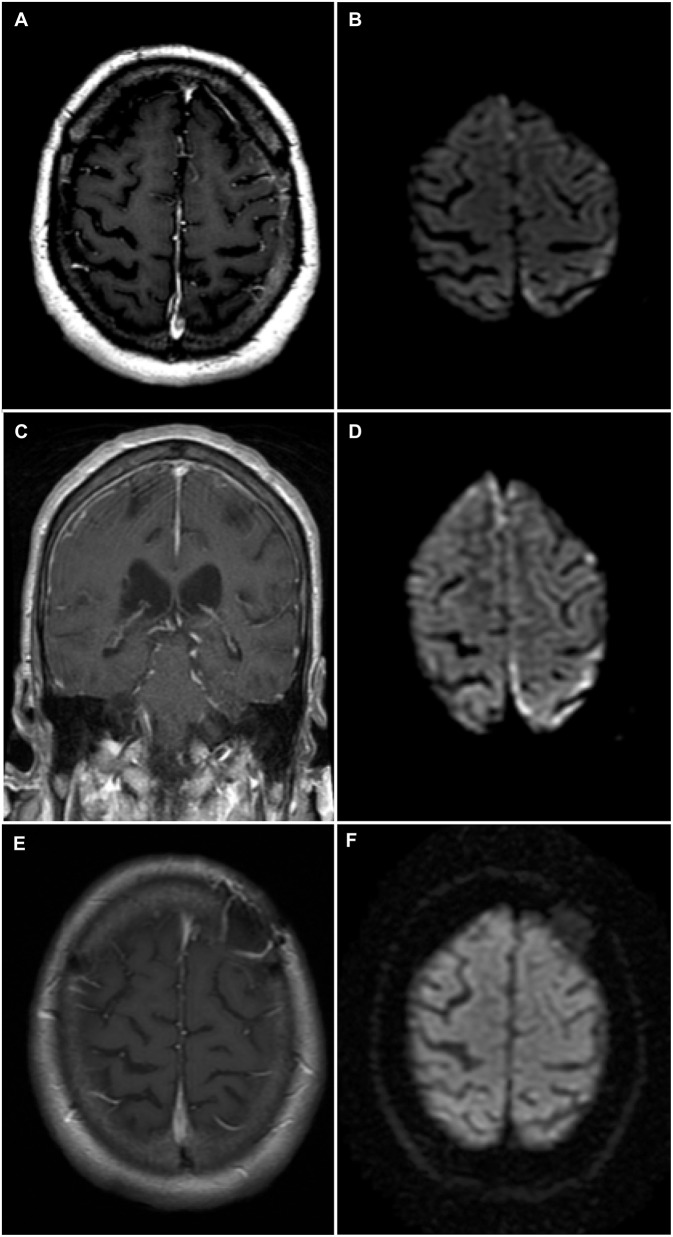
Brain magnetic resonance imaging (MRI)—Rheumatoid meningitis. **(A)** Axial T1-weighted sequence post-gadolinium shows faint contrast enhancement of the leptomeninges and underlying gyri over the left convexity. **(B)** Finite areas of diffusion restriction of the left parietal cortex near the vertex on axial diffusion weighted imaging (DWI) sequence. **(C)** Coronal T1-weighted sequence post-gadolinium shows longitudinal right frontal leptomeningeal and faint left leptomeningeal contrast enhancement. **(D)** Axial DWI sequence shows new areas of restricted diffusion in the right frontal parafalcine region along with increased volume of restricted diffusion in left parietal cortex near the vertex. **(E)** Axial T1-weighted sequence post-gadolinium obtained 3 months following immunosuppressive therapy showing no abnormal contrast enhancement, and left frontal postoperative changes. **(F)** Axial DWI sequence obtained 3 months following immunosuppressive therapy and demonstrating the resolution of previously documented findings.

A 74-year-old man was admitted to our institution for investigation of progressive neurological symptoms. The patient was diagnosed with seropositive RA in 2015, which was quiescent on maintenance methotrexate, hydroxychloroquine and low-dose prednisone (10 mg daily). Titers of both rheumatoid factor and antibodies to cyclic citrullinated peptide were elevated. One week prior to admission, the patient developed fluctuating confusion, apathy, word-finding difficulty, right-sided weakness and gait imbalance. He had also experienced several other similar self-limited episodes within the 3 preceding months. The initial neurological examination was remarkable for decreased attention span, severe expressive aphasia, bilateral postural tremor, right hemiparesis and hypoesthesia, as well as a shuffling and wide-based gait. Clinical evaluation by rheumatology confirmed absence of synovitis and no evidence of extra-articular RA involvement. Gadolinium-enhanced head magnetic resonance imaging (MRI) showed finite areas of scattered restricted diffusion and enhancement within the cortex and leptomeninges of the left hemisphere near the vertex, suspicious for meningoencephalitis (Figures 1A,B). Cerebrospinal fluid (CSF) analysis showed 6 white blood cells (WBCs), 85% lymphocytes and 15% monocytes, a protein concentration of 0.86 g/L (normal 0.15–0.45 g/L) and a normal glucose content. CSF bacterial and fungal cultures were negative, as were cryptococcal antigen, herpes simplex virus, and syphilis testing. Serum C-reactive protein was markedly elevated at 135 mg/L (normal 0–5 mg/L). Antinuclear antibodies and anti-neutrophil cytoplasmic antibodies were negative. Serologies for human immunodeficiency virus and anti-onconeural antibodies were negative. Methotrexate blood levels were within nontoxic range and immunoglobin G4-subclass titers were normal. Additionally, QuantiFERON-TB testing for *Mycobacterium tuberculosis* was negative. Serum angiotensin-converting enzyme (ACE) level was mildly elevated (66 U/L), as was beta 2-microglobulin (4.6 mg/L). High-resolution computed tomography (CT) scan of the chest was not suggestive of sarcoidosis.

Shortly after admission, the patient experienced two brief generalized tonic-clonic seizures. Following treatment with phenytoin, the patient's mental status and neurological examinations normalized completely. Electroencephalography (EEG) revealed nonspecific diffuse cortical slowing without interictal epileptiform activity. Two weeks later, the patient developed recurrence of his presenting neurological symptoms, in addition to new asymmetrical acute parkinsonism of the right hemibody (rigidity, bradykinesia, and resting tremor). Titration of his antiepileptic medication and addition of levetiracetam, lacosamide, and clobazam allowed for control of the symptoms, except for parkinsonism. The patient subsequently developed marked fluctuations of his mental status, ranging from an apathetic state to a confused and combative state. Repeat EEG and CSF analysis were essentially unchanged from previous. CSF cytology showed occasional atypical lymphocytes negative for CD3 and CD20. Additional analyses on CSF, including *Mycobacterium tuberculosis* culture, PCRs for Epstein-Barr virus and cytomegalovirus, ACE level and anti-neuronal cell surface antibodies, all proved negative. A follow-up MRI, 4 weeks after admission, showed progression of the left-sided cortical and leptomeningeal areas of restricted diffusion and enhancement, as well as new right frontoparietal cortical diffusion restriction and leptomeningeal enhancement (Figures 1C,D). A whole-body positron emission tomography scan did not reveal evidence of an underlying malignancy. Further work-up with a bone marrow biopsy showed no evidence of lymphoid neoplasm.

### Pathologic Findings

**Figure 2:**
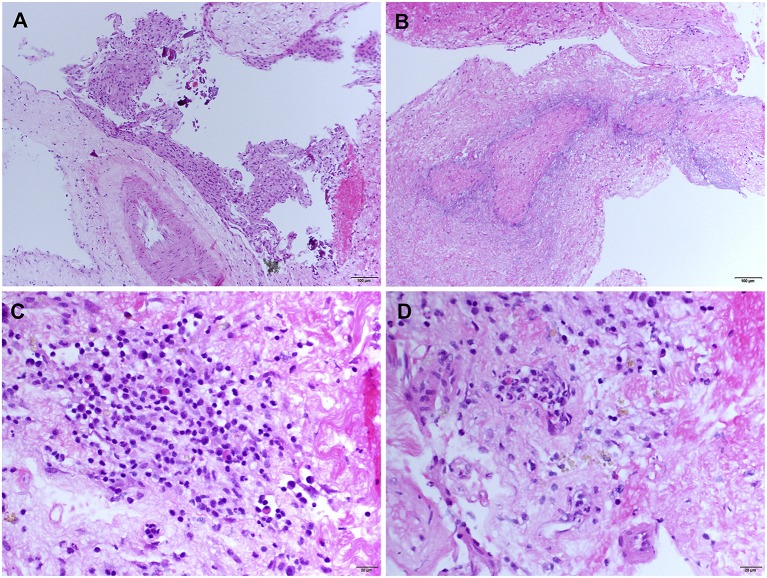
Meningeal histologic sections—Rheumatoid meningitis. Representative hematoxylin and eosin (H&E) stained sections. **(A)** Meningothelial hyperplasia (magnification 200). **(B)** Necrobiotic core surrounded by palisading macrophages (magnification 200). **(C)** Cluster of inflammatory infiltrate cells consisting mainly in small lymphocytes, mixed with few plasma cells and histiocytic cells (magnification 400). **(D)** Diffuse meningeal inflammatory infiltrate (magnification 400).

An open meningeal biopsy was performed and gross examination revealed thickening and opacification of the meninges. Hematoxylin and eosin (H&E) stained sections demonstrated meningothelial hyperplasia (Figure 2A) with acute and chronic inflammation associated with fibrosis and entrapment of the underlying brain parenchyma, which showed evidence of chronic gliosis. The most striking feature was the presence of classical zones of palisading necrobiosis (Figure 2B). The chronic inflammatory aggregates consisted in reactive CD3 positive T cells with fewer number of CD20 positive B lymphocytes, as well as CD68 positive macrophages and CD138 plasma cells with no evidence of light chain restriction (Figures 2C,D). Despite the presence of perivascular leptomeningeal inflammation, no significant vasculitis was present. All the special stains for microorganisms, mycobacteria, and fungal elements were negative. The histopathological findings were consistent with leptomeningeal involvement by nodular rheumatoid meningitis.

### Treatment and Outcome

Following histopathological confirmation of the diagnosis, immunosuppressive therapy with monthly cyclophosphamide (500–750 mg/m^2^ for 6 months) and high-dose corticosteroids was initiated. Corticosteroid regimen consisted of methylprednisolone 1,000 mg IV daily for 5 days, then prednisone 80 mg daily (1 mg/kg) tapered by 10 mg every 2 weeks up to a dose of 40 mg daily, at which point the dose was tapered by 5 mg every 2 weeks for 2 months then by 5 mg every 4 weeks for 4 months. Methotrexate was discontinued due to its failure to prevent disease progression, while hydroxychloroquine was continued. One month following treatment initiation, the patient's neurological examination improved, although confusion and bilateral postural tremor persisted. Furthermore, most parkinsonian features, except for mild leg rigidity, largely resolved following immunosuppressive therapy. Residual parkinsonism was not felt to be severe enough to warrant dopamine-replacement therapy, especially as it was felt that the parkinsonism was most likely secondary to the underlying RM and not due to a primary neurodegenerative process. Additionally, dopamine-replacement therapy was withheld as it was previously reported to be ineffective in a case of rheumatoid meningitis-induced parkinsonism (8). All antiepileptic drugs, aside from lacosamide, were eventually tapered without clinical or electrographic seizure recurrence. Repeat MRI 3 months after treatment initiation showed complete resolution of previous findings (Figures 1E,F), correlating with normalization of CRP levels. Despite this radiographic improvement, the patient suffered from persistent behavioral and cognitive deficits with intermittent periods of agitation. The neuropsychiatric sequelae remained disabling enough for the patient to eventually be transitioned to a long-term care facility.

## Discussion

Three main histopathological findings characteristic of rheumatoid meningitis may be observed on pathologic examination of the meninges, including nonspecific inflammation, rheumatoid nodules and vasculitis (2). Only in rare instances (<15% of cases) are the three features described in association (Table 1). Meningeal inflammation, involving the pachymeninges and/or the leptomeninges, is the most common pathological feature, found in more than 95% of cases. The inflammatory infiltrate consists primarily in mononuclear cells, particularly plasma cells, and multinucleated giant cells (2). Focal areas of necrosis and polymorphonuclear cells are seldom encountered. The predominance of plasma cell-rich reaction appears relatively specific for rheumatoid meningitis and a distinguishing factor from other connective tissue disorders (3). Meningeal rheumatoid granulomas, observed in about 40% of cases (Table 1), are histologically identical to subcutaneous nodules and include a central necrotic core surrounded by palisading macrophages and lymphocytes. Aside from the cranial meninges, rheumatoid nodules may be found in choroid plexus and spinal meninges in <10% of cases. Vasculitis is the least common of the pathological findings, with <20% of patients being affected (Table 1), and tends to affect predominantly small meningeal and parenchymal vessels (11). Vessel wall infiltrate consists in a lymphoplasmacytic reaction (2). Rarely, spinal parenchymatous vasculitis and basal ganglia vasculitis have been reported in the setting of rheumatoid meningitis (37).

**Table 1:** Pathological findings, treatment regimens, and outcome of rheumatoid meningitis cases reported since 2005.

**Authors**	**Year**	**Patient (Age, sex)**	**Pathological findings**			**Anti-rheumatic drugs prior to diagnosis of RM**	**Treatment regimen**	**Outcome**
			**Meningeal inflammation**	**Rheumatoid nodule**	**Vasculitis**			
Chowdhry et al. (10)	2005	78F	+	+	−	CS, MTX, leflunomide	CS	Improvement
Jones et al. (11)	2006	58F	+	−	−	None	CS, CYC	Improvement
Ahmed et al. (12)	2006	77M	+	−	−	MTX, HCQ, sulfasalazine, minocycline, leflunomide	CS	Improvement
Chou et al. (13)	2006	58F	+	−	−	None	CS, CYC, infliximab	Improvement, then relapsed, then improvement
Starosta et al. (14)	2007	67M	+	+	−	None	CS, MTX	Incomplete improvement
	2007	76F	+	+	−	CS, MTX, infliximab	CS	Incomplete improvement
Schmid et al. (15)	2009	64M	+	−	+	MTX, infiximab	CS, rituximab, d/c infliximab	Improvement
Shimada et al. (16)	2009	53F	+	−	−	CS, MTX	CS	Incomplete improvement
Ii et al. (17)	2009	68M	+	−	−	None	CS	Improvement
Koide et al. (18)	2009	74F	+	−	−	Not reported	CS	Improvement
Luessi et al. (19)	2009	64F	+	+	+	MTX, azulfidine	CS, AZA, CYC, infliximab, MTX	Worsening
Cianfoni et al. (20)	2010	74F	+	+	−	CS, MTX	CS, IT MTX	Worsening
Matsushima et al. (21)	2010	80F	+	+	+	CS, etanercept, bucillamine, sulfasalazine	CS	Improvement
Servioli et al. (3)	2011	80F	+	−	−	CS, HCQ	Not reported	Not reported
Kim et al. (22)	2011	66M	+	−	+	None	CS	Improvement
Huys et al. (23)	2012	58F	+	+	−	MTX, adalimumab	CS, rituximab, leflunomide, d/c MTX, d/c adalimumab	Improvement
Duray et al. (24)	2012	73M	+	+	−	CS, MTX	CS, CYC	Improvement
Krysl et al. (25)	2013	62M	+	−	−	Chloroquine	CS	Improvement
Hayashi et al. (8)	2014	68M	+	−	−	CS	CS	Incomplete improvement
Bourgeois et al. (5)	2014	70M	+	−	−	Not reported	CS, HCQ, sulfasalazine	Improvement
Rijkers et al. (26)	2014	56F	−	+	−	Not reported	CS	Incomplete improvement
Roy et al. (7)	2015	50F	+	−	−	MTX, sulfasalazine	CS, MMF, d/c MTX	Improvement
Lu et al. (27)	2015	60F	+	+	−	CS, auranofin	CS	Improvement
Seago et al. (28)	2016	66F	+	−	−	Infliximab	CS	Improvement
Nihat et al. (29)	2016	71F	+	+	+	Adalimumab, MTX	CS, CYC, MTX	Improvement
Magaki et al. (4)	2016	37M	+	−	−	None	CS	Improvement
Matsuda et al. (30)	2016	66M	NR	NR	NR	CS, MTX, iguratimod	CS, d/c MTX	Improvement
Moeyersoons et al. (31)	2017	49M	NR	NR	NR	Adalimumab, leflunomide	CS, rituximab, d/c adalimumab, d/c leflunomide	Improvement
Tsuzaki et al. (32)	2017	65M	+	−	−	CS, MTX, etanercept	CS, tocilizumab, d/c etanercept	Improvement
Degboé et al. (33)	2017	59M	+	−	−	MTX	CS, rituximab	Improvement
Jessee et al. (34)	2017	68F	+	−	−	None	CS, MTX	Incomplete improvement
Choi et al. (35)	2017	65F	+	−	+	CS, MTX, leflunomide	CS	Improvement
Parsons et al. (6)	2018	76M	+	−	−	MTX	CS, MTX	Improvement
Alexander et al. (36)	2018	73M	+	−	−	Leflunomide	CS, rituximab	Incomplete improvement
This report	2018	74M	+	+	−	CS, MTX, HCQ	CS, CYC, d/c MTX	Incomplete improvement

*Review of the 35 cases of rheumatoid meningitis published since 2005, and excluding cases in which no information regarding treatment and outcome was available. Unless otherwise specified, the anti-rheumatic drugs on presentation were continued after diagnosis of rheumatoid meningitis. The mean age at presentation was 65 years (± 9.6 years of standard deviation). AZA, azathioprine; CS, corticosteroids; CYC, cyclophosphamide; D/C, discontinued; F, female; HCQ, hydroxychloroquine; IT, intrathecal; M, male; MMF, mycophenolate mofetil; MTX, methotrexate; NR, not reported; RM, rheumatoid meningitis*.

Although the semiology of the presenting neurological signs and symptoms of our patient (mainly expressive aphasia, right hemiparesis and hypoesthesia) was not suggestive of a clinical seizure, the normalization of the neurological examination following treatment with antiepileptics points toward an underlying epileptic phenomenon. However, multiple EEGs recorded while the patient was symptomatic failed to show epileptiform activity. Alternatively, short unwitnessed seizures may have triggered prolonged waves of cortical spreading depression. Akin to our observations, Chowdhry et al. (10) described a patient who presented with multiple 30 min spells of left-sided paresthesia and hemiparesis, the frequency and severity of which decreased following treatment with phenytoin. Again, such a presentation points toward an epileptic phenomenon. Interestingly, the EEG of this patient also failed to show epileptiform activity.

Rheumatoid meningitis may rarely mimic Parkinson's disease (8) and progressive supranuclear palsy (9). Further adding to the atypical presentation of the case is the development of acute parkinsonism by our patient. Acute-onset parkinsonism is a rare occurrence most often attributable to medication or infectious cause (38), and had yet to be described in rheumatoid meningitis. Vasculitic involvement of the basal ganglia is the purported causative mechanism of rheumatoid meningitis-related extrapyramidal syndromes (9, 37). Although deep cerebral biopsy was not performed in our patient, one may hypothesize that our patient might have had vasculitic involvement of the basal ganglia, especially since alternative causes of parkinsonism were excluded and that parkinsonian features greatly improved following immunosuppressive therapy.

As was the case with our patient, there is often a significant delay between symptom onset and diagnosis of rheumatoid meningitis. This lag period may be critical for achieving optimal clinical outcomes, and may account for our patient's incomplete recovery, despite aggressive immunosuppressive therapy, and raises the key question as to whether earlier recognition and treatment of the condition would have yielded better outcomes.

Therapeutic management of rheumatoid meningitis remains a major challenge, and the optimal immunosuppressive regimen has yet to emerge. Corticosteroids have been the cornerstone of treatment since cases of rheumatoid meningitis began to be described. The first extensive case series from 1989 described 19 cases (2). Of these, only nine patients were treated with steroids, and one also received cyclophosphamide. Maximum survival was 4 years with death occurring in more than 80% of patients. Most of these cases were diagnosed on autopsy and were likely undiagnosed when symptomatic thus preventing treatment. A review (39) performed 14 years later revealed that most cases were still being treated only with steroids although survival slightly improved, likely due to improved diagnostic accuracy. Our review of cases published since 2005 (total of 35 cases, including the patient described in this report) reveals a noticeable increase in use of combination therapy and steroid-sparing strategies (56% for combination therapy vs. 44% corticosteroids monotherapy), which may be accounted for by the previously described elevated death rate in patients treated with corticosteroids alone (>60%). Dosing of steroids has been extremely variable and is unlikely a major determinant of the effectiveness of therapy. Although typically given via pulse regimens, one author reported remission with a dosing of prednisone 60 mg (10). Interestingly, the complete response rate in our series was almost identical for patients treated with combination therapy and monotherapy (around 70% in both groups), although this assessment is likely inaccurate owing to the often incomplete follow-up information provided in the previously published reports. Increased awareness of the condition by clinicians, improved diagnostic accuracy and prompt initiation of treatment are all potential factors to account for the similar response rate. Furthermore, vasculitis has often been considered to be of poor prognostic significance, portending a high failure rate to therapy. This trend was not confirmed in this series, where the response rate was around 80%. Six cases describing evidence of CNS rheumatoid vasculitis were found, of which three were treated with corticosteroids alone and three with combination immunosuppressive therapy. While the complete response rate was 100% in the first subgroup, it was only 66% in the second (Table 1).

Cyclophosphamide emerged as the most commonly used immunosuppressive agent likely due to its established use in CNS manifestations of systemic rheumatic diseases. However, recurrences in patients treated with this agent have occurred. One patient received both intravenous (9 months) and oral (2 months) courses yet his disease remained active (19). Another report described the resolution of symptoms after cyclophosphamide use, with recurrence 4 months later and after three infusions of infliximab, with subsequent resolution following a second course of cyclophosphamide (13). It is unclear whether this was a failure of cyclophosphamide or disease “reactivation” by the tumor necrosis factor α (TNFα) inhibitor, as discussed later.

The anti-CD20 monoclonal antibody rituximab has also been used to treat rheumatoid meningitis and may offer a therapeutic alternative to cyclophosphamide. Its first reported use dates back to 2009 (15) and coincides with its emergence as an effective agent in RA. The rationale behind its use was based on presence of a high percentage of CD20-expressing B lymphocytes in meningeal specimens of individuals affected by rheumatoid meningitis (15). Its advantages over cyclophosphamide include ease of use and a lesser side effect profile. To date, in the five cases where rituximab was used, authors did not report disease recurrence during follow-up periods ranging from 6 months to 2 years. This agent was used as monotherapy in two cases but was also combined with leflunomide and methotrexate in the other three cases in order to prevent auto-antibody formation against rituximab (23) or as a steroid-sparing agent (15, 33).

On the other hand, treatment with TNFα inhibitors might be ineffective. Several case reports mention that rheumatoid meningitis occurred in patients who already were on TNFα inhibitors. Interestingly, anti-TNFα agents have been associated with the development of accelerated nodulosis (40) and two reports present cases of aseptic meningitis in the context of adalimumab (41) and etanercept (42) use. There are at least six cases of meningeal rheumatoid disease occurring in patients being treated with anti-TNFα agents reported in the literature that implicate infliximab (13, 15, 28), adalimumab (12, 23), and etanercept (32). It remains unclear whether this is confounded by disease severity or whether these agents were simply non-protective due to low blood-brain-barrier permeability (13). There has also been no reported case of RM treated with TNFα inhibitor monotherapy. Therefore, until further evidence emerge, it may be prudent to avoid using anti-TNFα agents to treat rheumatoid meningitis.

Methotrexate-induced accelerated nodulosis is a known side effect of longstanding treatment for RA and tends to affect small joints (43). Anecdotal evidence has also suggested that methotrexate may predispose to development of meningeal nodules (44, 45). Several cases of nodular meningitis have been reported in patients on long-term methotrexate therapy for RA (7, 15, 19, 20, 23). However, some patients eventually improved clinically while being kept on methotrexate. Occurrence of breakthrough rheumatoid meningitis on methotrexate, as in our patient, may reflect its limited efficacy for this condition, which may be accounted for by its poor blood-brain-barrier permeability (46). It must also be noted that in the literature describing the utilization of methotrexate as a therapy for rheumatoid meningitis, this drug was always combined with another immunosuppressive agent (9, 14, 15, 19, 33). Available evidence thus preclude any conclusion on the efficacy of methotrexate alone in the treatment of rheumatoid meningitis.

## Conclusion

Rheumatoid meningitis has historically been ascribed a high morbidity and mortality, especially when associated with CNS vasculitis. Debate is still ongoing regarding the optimal therapeutic strategy for this condition. Our review of the literature, focusing on recently published cases, indicates that there has been a definite improvement of morbidity and especially mortality associated with this condition, likely owing to improved diagnostic accuracy and prompt aggressive immunosuppressive treatment initiation. Rituximab appears to be a particularly promising option, as no treatment failure has been observed following its use.

## Ethics Statement

Clinical data in this case report was collected with the consent of the patient. A written informed consent was obtained from the patient for the publication of this case report. The case report is exempt from institutional review board approval.

## Author Contributions

DP and MW contributed to manuscript preparation and background research. M-CG contributed to pathology interpretation, manuscript preparation, and reviewing. HA, AB, JK, EV, A-LL, and SL contributed to manuscript preparation and reviewing. All authors read and approved the manuscript.

### Conflict of Interest Statement

The authors declare that the research was conducted in the absence of any commercial or financial relationships that could be construed as a potential conflict of interest.
